# Proliferation and T_H_1/T_H_2 Cytokine Production in Human Peripheral Blood Mononuclear Cells after Treatment with Cypermethrin and Mancozeb *In Vitro*

**DOI:** 10.1155/2014/308286

**Published:** 2014-09-18

**Authors:** Rajesh Mandarapu, Rajanna Ajumeera, Vijayalakshmi Venkatesan, Balakrishna Murthy Prakhya

**Affiliations:** ^1^International Institute of Biotechnology and Toxicology, Kancheepuram District, Padappai, Tamil Nadu 601301, India; ^2^National Institute of Nutrition (ICMR), Jamai Osmania, Hyderabad, Telagana 500 007, India

## Abstract

In recent times, human cell-based assays are gaining attention in assessments of immunomodulatory effects of chemicals. In the study here, the possible effects of cypermethrin and mancozeb on lymphocyte proliferation and proinflammatory (tumor necrosis factor (TNF-) *α*) and immunoregulatory cytokine (interferon- (IFN-) *γ*, interleukins (IL) 2, 4, 6, and 10) formation *in vitro* were investigated. Human peripheral blood mononuclear cells (PBMC) were isolated and exposed for 6 hr to noncytotoxic doses (0.45–30 *µ*M) of cypermethrin or mancozeb in the presence of activating rat S9 fraction. Cultures were then further incubated for 48 or 72 hr in fresh medium containing phytohemagglutinin (10 *µ*g/mL) to assess, respectively, effects on cell proliferation (BrdU-ELISA method) and cytokine formation (flow cytometric bead immunoassays). Mancozeb induced dose-dependent increases in lymphocyte proliferation, inhibition of production of TNF*α* and the T_H_2 cytokines IL-6 and IL-10, and an increase in IFN*γ* (T_H_1 cytokine) production (at least 2-fold compared to control); mancozeb also induced inhibition of IL-4 (T_H_2) and stimulated IL-2 (T_H_1) production, albeit only in dose-related manners for each. In contrast, cypermethrin exposure did not cause significant effects on proliferation or cytokine profiles. Further studies are needed to better understand the functional significance of our *in vitro* findings.

## 1. Introduction

Pesticide-associated immune dysfunction has gained regulatory and public attention in the past 20 years due to the wide use of these agents in agriculture, industries, and domestic purposes. Chronic exposure to pesticides increases the risk of immunomodulation [1–5] and the onset of lymphoid neoplasms [6] and leukemias [7]. Cypermethrin and mancozeb are widely used in agriculture, households, and industries due to their “low” toxicity in mammals and short environmental persistence [8–11]. Earlier studies have indicated the immunomodulatory effects such as decrease in antiovalbumin titer of blood sera, autologous rosette formation of T-lymphocytes [12–14], lymphocyte transformation rate, and an increase in neutrophil phagocytosis rate [15] from exposure to cypermethrin in animal models. Similarly, occupational exposure to mancozeb had shown significant increase in T-cell functional response such as mitogen-induced proliferation and a decrease in TNF-alpha [16, 17]. Although, this information is limited to few functional properties, it clearly indicates that the proliferation and T_H_1/T_H_2 cytokine production, the key indicators, play a major role in etiology of several immunological disorders.

There is a wide range of experimental protocols and guidelines that have been validated for assessing the chemical induced immune dysfunctions. These assays include local lymph node assay (LLNA), guinea pig maximization test (GPMT) for the dermal allergic potential, T-cell dependent antibody response (TDAR) assay for immunosuppression, and popliteal lymph node assay (PLNA) for autoimmune reactions. Nevertheless, these assays are based on animal models and having limitations such as extrapolating the animal data to humans, false positives or negatives, and several others in evaluating the immune system. Furthermore, the 3Rs (reduce, refine, and replace) strongly recommend using alternative approaches for evaluating the immunocompetence of chemicals and xenobiotics.

Proliferation and cytokine production are two key functions of immune cells and, as such, are important endpoints to examine during any evaluation of immunotoxic potential of a given xenobiotic [18–20]. Mitogen-induced proliferation of lymphocytes is often a preferable assay that correlates with the status of cell-mediated immunity in a host [21] after exposure to a xenobiotic. Similarly, cytokines, as regulators of immune function, are sensitive indicators of immunomodulation in an exposed host [22–25].

As immune functions are mediated by several cytokines/chemokines (that, in turn, are influenced by bacterial or viral infections, drugs, and/or exposure to environmental or workplace agents), it is necessary to analyze a panel of cytokines to better understand effects of a given toxicant on host immunocompetence [26]. Evaluations of proinflammatory and immunoregulatory agents also provide valuable information during assessments of chemical induced immunomodulation. In addition, because maintaining homeostasis of T-helper (T_H_) type 1 and T_H_2 cytokines is critical to immunocompetence [27, 28], deviations in levels of either of these or the balance between these two can provide strong evidence to understand immunopathologies induced by chemical exposures [24] and also help in developing better testing regimens for evaluating the immune system.

The present study was designed to evaluate the potential effects of two widely used pesticides, cypermethrin and mancozeb, on functional properties of immune system through lymphocyte proliferation and T_H_1/T_H_2 cytokine production in human PBMC.

## 2. Materials and Methods

### 2.1. Chemicals

Cypermethrin (>99%) and mancozeb (>95%) were purchased from Sigma (St. Louis, MO). Stock solutions of these chemicals were made in dimethyl sulfoxide (DMSO; Sigma) and stored at −80°C. Stock solutions were diluted in DMSO at the desired concentrations before further dilution in culture medium. Final culture levels of DMSO never exceeded 0.1%.

### 2.2. Peripheral Blood Mononuclear Cells

Upon obtaining informed consent, peripheral blood was collected from individual male volunteers 26–35 years of age. Selection of volunteers was based on the criteria listed by Indian Council of Medical Research (ICMR-2004), briefly, only those volunteers who are nonsmokers, are nonalcoholics, and have had no recent history of illness as certified by a medical practitioner. The study was approved by the Institutional Ethics Committee of the International Institute of Biotechnology and Toxicology, in its meeting held on April 2, 2011.

Blood was collected into heparinized tubes and mononuclear cells were isolated using Ficoll-Hypaque (*ρ* = 1.077 g/mL) density gradient centrifugation at 400 ×g for 30 min. The buffy coat containing mononuclear cells was isolated, transferred to a fresh centrifuge tube, and washed twice with PBS (using ≈3 vol of collected buffy coat each time). The final cell pellet containing peripheral blood mononuclear cells (PBMC) was resuspended to a final level of 1-2 × 10^6^ cells/mL in RPMI 1640 medium supplemented with L-glutamine, 10% heat-inactivated fetal bovine serum, 100 U penicillin/mL, and 0.1 mg streptomycin/mL (all Gibco, Paisley, UK).

### 2.3. Cytotoxicity Assessment

Preliminary cytotoxicity studies were performed using PBMC from 2 donors/test agent to assess biovariance. PBMC (10^5^ cells/well, 24-well plate) were exposed for 24 hr to serial doses of cypermethrin or mancozeb in the presence of a metabolic activator (i.e., rat liver S9 fraction (Moltox, Boone, NC)). Immediately before use, 10% S9 mix containing 15% S9 fraction was added to the reaction medium. Based on trypan blue (Gibco, Paisley, UK) dye exclusion, doses that caused >10% of cytotoxicity were excluded from further analysis.

### 2.4. Culture Set-Up/PBMC Exposure

To assess biological variance, assays were performed (in triplicate) using PBMC from three donors with each chemical separately. Cultures of PBMC (10^5^ cells/well, 24-well plate) were exposed to noncytotoxic doses of cypermethrin (1.87, 3.75, 7.50, 15, or 30 *μ*M) or mancozeb (0.45, 0.93, 1.87, 3.75, or 7.50 *μ*M) or to medium containing solvent (DMSO) or medium alone for 6 hr. All cultures contain freshly prepared S9 mix. At the end of the exposure, the medium was removed from each well and fresh medium containing phytohemagglutinin (10 *μ*g PHA/mL; Gibco) mitogen was added. The cells were then incubated at 37°C under 5% CO_2_ and 95% humidity for 48 or 72 hr to assess, respectively, cell proliferation and cytokine release.

### 2.5. Cell Proliferation-BrdU ELISA Method

Cell proliferation was measured upon completion of the 48 hr incubation period as noted above using a BrdU-ELISA kit (Roche Diagnostics, Mannheim, Germany) as per manufacturer instructions. In brief, kit-provided BrdU labeling solution (20 *μ*L) was added to each well and the plate was incubated at 37°C overnight. Thereafter, the cells were centrifuged at 300 ×g for 10 min, the labeling solution was removed, and the plate then was dried at 60°C for 1 hr before the cells were fixed by addition of FixDenat (200 *μ*L/well) solution and incubation at room temperature for 15 min. Antibody conjugate (anti-BrdU-POD solution, 100 *μ*L/well) was then added and the plate was incubated at room temperature for 90 min. The cells were then washed twice with PBS (200 *μ*L) and kit-provided substrate solution (100 *μ*L) was added to each well. The plates were left at room temperature for 20 min and the absorbance in each well was then measured at 370 nm in an automated plate reader (Awareness Technology, Inc., Palm city, FL). Sets of blank (100 *μ*L culture medium alone) and control wells were included in each experiment.

### 2.6. T_H_1 and T_H_2 Cytokine Analysis

After the 72 hr incubation with PHA, culture supernatants from each well were harvested and stored at −80°C until analysis. Levels of select proinflammatory (tumor necrosis factor (TNF)-α) and immunoregulatory (T_H_1/T_H_2) cytokines (e.g., interferon- (IFN-) *γ*, interleukin (IL-) 2, IL-4, IL-6, and IL-10) in samples were estimated using a flow cytometric bead immunoassay (according to manufacturer protocols) in a FACSAria-II system (BD Biosciences, San Jose, CA). The cytometer was calibrated using standard beads before analysis and standard curves were generated for each cytokine using known concentrations (provided in kit) ranging from 20 to 5000 pg/mL.

### 2.7. Data Analysis

Cell proliferation was expressed in terms of percentage growth, with 100% corresponding to the values seen with the control wells. Triplicate data was averaged and expressed as mean ± SE for three individual experiments (*n* = 3) conducted from PBMC of three different donors for each compound. Cytokine analyses were performed using FCAP Array software (version 3.0). The concentration of each analyte in a sample was extrapolated from a calibration curve generated in parallel and that was modeled by a five-parameter log-logistic curve (5PL) for each analyte, against log-transformed median fluorescence intensity (MFI) versus concentrations.

Cytokine quantities obtained with the FCAP Array software were compared with concurrent solvent controls and statistical analysis performed by two-way ANOVA followed by a Newman-Keuls Test post hoccomparison using Prism software (v6.03 for Windows, GraphPad, San Diego, CA). For all of the comparisons, a 0.05 *α*-type error (*P* < 0.05) was considered as significant.

## 3. Results

### 3.1. Effects on Proliferation

**Figure 1:**
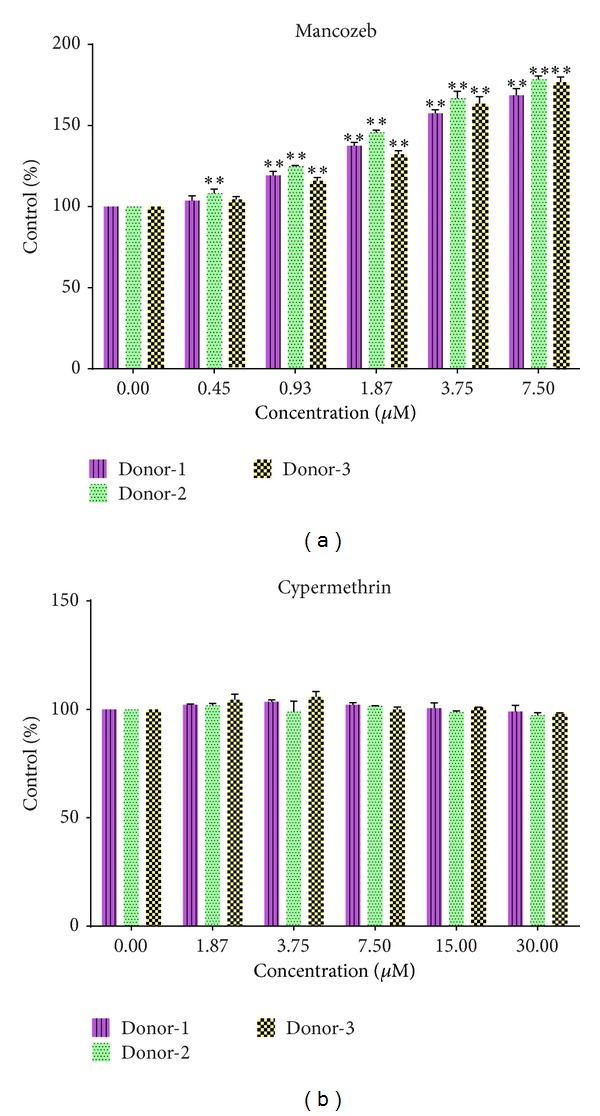
Proliferation-BrdU ELISA assay. Proliferation rate in mitogen (PHA-) stimulated PBMC cultures. (a) Mancozeb; (b) cypermethrin. ∗*P* < 0.05; ∗∗*P* < 0.01 versus zero control. For each donor, statistical analyses performed across the doses showed that values were consistently significantly different as a function of dose in the mancozeb study.

A dramatic effect on PHA-induced lymphocyte proliferation was observed with mancozeb; the percentage proliferation (compared to that seen with the solvent control) was increased in a dose-dependent manner at the doses tested (0.45–7.5 *μ*M; Figure 1(a)). At 7.5 *μ*M, the average value (as percent of control) had reached 174.5 (±5.2)% (Table 1). In contrast, cypermethrin caused nominal nonsignificant changes in proliferation at the doses tested (1.87–30 *μ*M; Figure 1(b)) relative to the control values.

### 3.2. Effect on Proinflammatory TNFα Production

**Figure 2:**
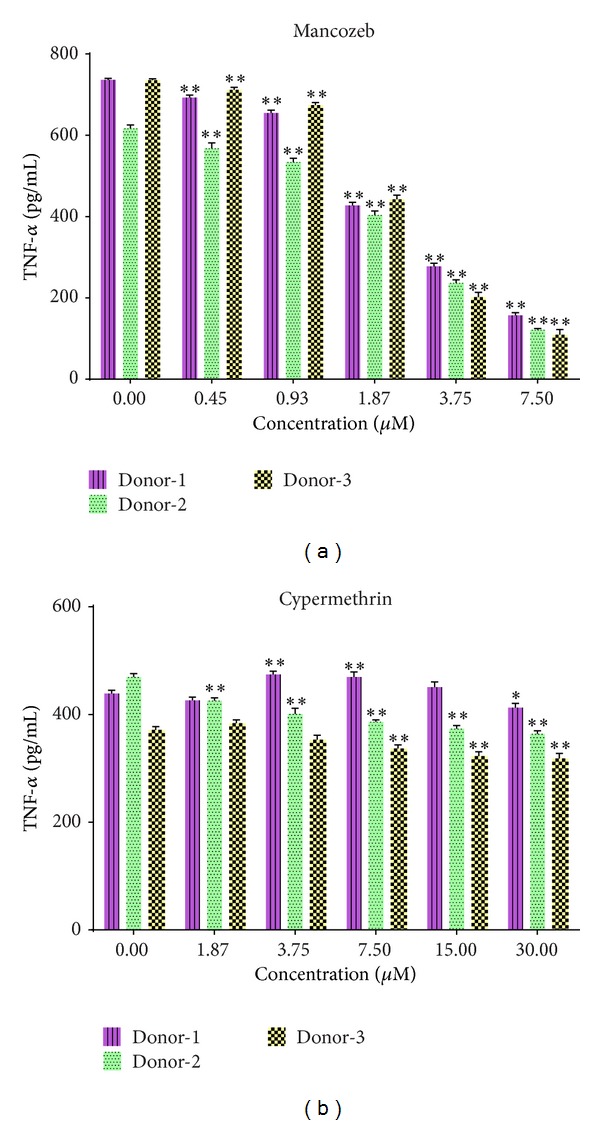
Proinflammatory response. Effects on levels of proinflammatory cytokine TNFα in culture supernatants of PBMC. (a) Mancozeb. (b) Cypermethrin. Values shown are mean (±SE) from triplicate samples conducted with PBMC from three volunteers. ∗*P* < 0.05; ∗∗*P* < 0.01 versus zero control. For each donor, statistical analyses performed across the doses showed that values were consistently significantly different as a function of dose in the mancozeb study.

Dose-dependent decreases in PHA-stimulated TNFα release were evident in the mancozeb-exposed cultures (Figure 2(a)). Maximal inhibition of TNFα production was seen at the highest test dose and 7.5 *μ*M (average of the three donor populations = 129.1 (±25.2) pg/mL; Table 1) was more than 2-fold below the control culture levels (average = 696.4 (±60.2) pg/mL; Table 1). Although slight changes were observed in the cypermethrin-exposed cultures, these were very modest even at the highest test dose, 30 *μ*M (average = 365.1 (±42.5) pg/mL; Table 1) relative to the control value (average = 426.6 (±44.4) pg/mL; Table 1) (Figure 2(b)).

### 3.3. Effect on T_H_1 (IL-2 and IFN*γ*) Cytokines

**Figure 3:**
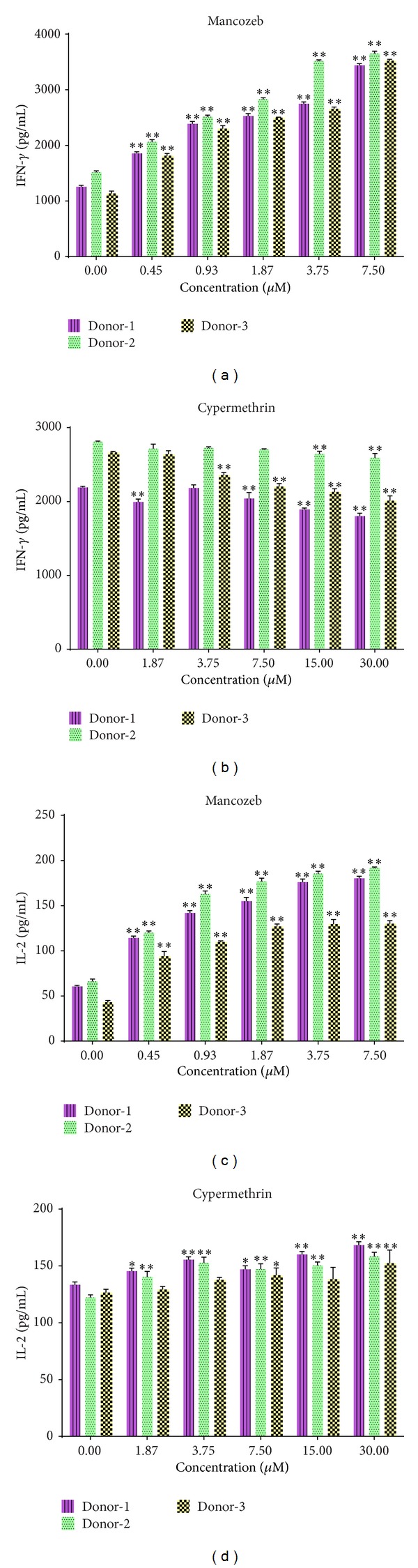
T_H_1 cytokine profiles. Effect on T_H_1 cytokines IFN*γ* and IL-2 production in PBMC cultures. (a) Mancozeb IFN*γ*. (b) Cypermethrin IFN*γ*. (c) Mancozeb IL-2. (d) Cypermethrin IL-2. Values shown are mean (±SE) from triplicate samples conducted with PBMC from three volunteers. ∗*P* < 0.05; ∗∗*P* < 0.01 versus zero control. For each donor, statistical analyses performed across the doses showed that resultant IFN*γ* values were consistently significantly different as a function of dose in the mancozeb study.

Mancozeb induced formation and release of both IL-2 and IFN*γ* by the PBMC. Maximal induction of cytokine production (a 2-fold change) was observed at highest concentration, that is, 7.5 *μ*M. IFN*γ* production increased dose-dependently to (average) 3539.2 (±103.7) pg/mL from 1303.4 (±175.1) pg/mL for the control cells (Table 1, Figure 3(a)); IL-2 levels increased (albeit in non-dose-dependent manner) to 167.4 (±28.6) pg/mL from 56.6 (±10.9) pg/mL (Table 1, Figure 3(c)). Changes in levels of these T_H_1 cytokines in the cypermethrin-exposed cultures were weak relative to control cell values. Interestingly, IFN*γ* production seemed to decrease, albeit insignificantly, from (average) 2555.9 (±281.0) pg/mL down to 2134.9 (±364.2) pg/mL at the 30 *μ*M dose (Table 1, Figure 3(b)); IL-2 production increased from 127.4 (±6.2) pg/mL to 159.6 (±12.8) pg/mL (Table 1, Figure 3(d)).

### 3.4. Effect on T_H_2 (IL-4, IL-6, and IL-10) Cytokines

**Figure 4:**
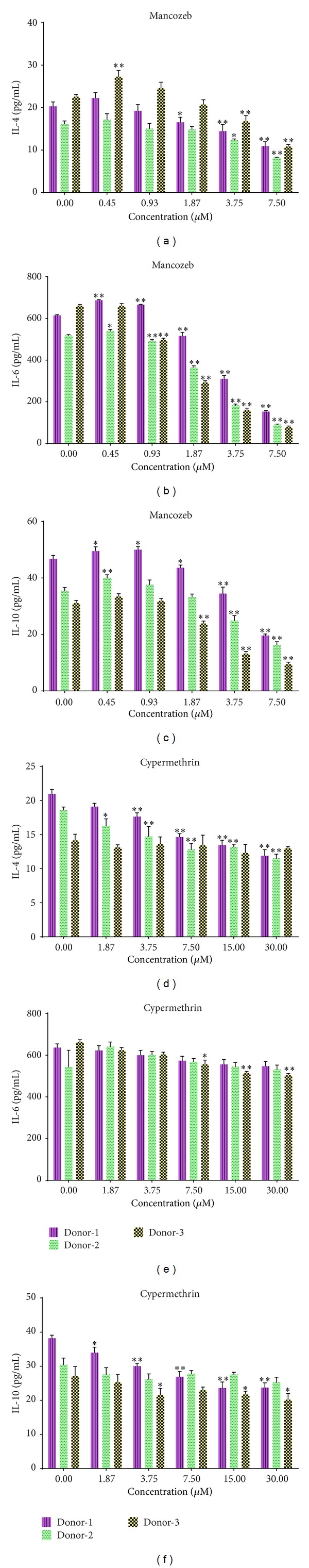
T_H_2 cytokine profiles. Effect on T_H_2 cytokines IL-4, IL-6, and IL-10 production in PBMC cultures. Mancozeb: (a) IL-4; (b) IL-6; and (c) IL-10. Cypermethrin: (d) IL-4; (e) IL-6; and (f) IL-10. Values shown are mean (±SE) from triplicate samples conducted with PBMC from three volunteers. ∗*P* < 0.05; ∗∗*P* < 0.01 versus zero control. For each donor, statistical analyses performed across the doses showed that resultant IL-6 values were consistently significantly different as a function of dose in the mancozeb study. Further, in that same study, IL-10 values were also consistently significantly different as a function of dose for doses of ≥0.93 *μ*M.

T_H_2 cytokine production by PBMC was significantly inhibited with increasing doses of mancozeb. Maximal inhibition of cytokine production was observed at highest 7.5 *μ*M concentration, with IL-4 production dropping to (average) 10.0 (±1.7) pg/mL from 19.7 (±3.1) pg/mL (Figure 4(a), Table 1), IL-6 (dose-dependently) to 107.7 (±34.9) pg/mL from 597.8 (±63.6) pg/mL (Figure 4(b), Table 1), and IL-10 (dose-dependently) to 15.1 (±4.6) pg/mL from 37.8 (±7.2) pg/mL (Figure 4(c), Table 1). On the other hand, while there were changes in IL-4 levels with at least two donor PBMC populations exposed to cypermethrin, the average values at 30 *μ*M were comparable to the controls, though the levels had declined from 17.9 pg IL-4/mL (±3.2) to 12.1 (±1.2) pg/mL (Figure 4(d), Table 1). While the impact from this pesticide on IL-6 production by the cells was slight IL-6 levels dropped from 614.5 (±90.8) pg/mL to 527.3 (±34.1) pg/mL; (Figure 4(e), Table 1), a clear trend toward inhibition of IL-10 formation was evident for at least two donor PBMC populations. However, the average values at 30 *μ*M were not significantly different from the control (31.9 (±5.8) pg/mL versus 23.0 (±3.3) pg/mL; Figure 4(f), Table 1).

## 4. Discussion

Exposures to pesticides as environmental contaminants or in the workplace elicit a wide variety of adverse effects on the human immune system and often lead to various immune-based/related disorders [2, 29]. Despite the number of studies available for assessing the immune functions, in recent years, there has been a growing interest in considering the role of proinflammatory and immunoregulatory cytokines in toxic responses to chemical exposure; this has, in turn, resulted in an increased consideration in measures of these parameters when developing risk assessment strategies [30]. Homeostasis of the levels of T_H_1 and T_H_2, as well as proinflammatory, cytokines is crucial. Deviations in levels of either of these or the balance between these two can provide strong evidence to understand immunopathologies induced by chemical exposures [24, 31].

Immunomodulatory effects of two widely used pesticides, that is, cypermethrin and mancozeb, in PBMC were evaluated in the present study. The* in vitro* data here indicated that mancozeb exerted potential immunomodulatory effects characterized by (i) increased cell proliferative responses to PHA stimulation; (ii) significant reductions in PHA-induced TNFα release; and (iii) significant alterations in T_H_1 and T_H_2 cytokine profiles. These outcomes reflect the same types of effects of mancozeb on mitogen-induced proliferation and on TNFα and IL-2 levels that had been reported earlier [16, 17, 32, 33]. On the other hand, here, cypermethrin only caused slight changes in the cytokine profiles or on mitogen-induced proliferation.

Numerous* in vivo* studies have reported dose-dependent decreases in hematologic endpoints, such as erythrocyte counts and packed cell volumes that were accompanied by significant changes in lymphocyte, monocyte, and total leukocyte counts as a result of exposure to cypermethrin [34–36] or mancozeb [17, 37]. The results in the present* in vitro *study correlated fairly well with those* in vivo* findings and indicated that the mancozeb directly affected immune cells. However, cypermethrin exposure did not show any effect on lymphocyte proliferation and cytokine production. This clearly shows that the pesticides will act differently on immune cells and have different effects.

The range of* in vitro* doses tested here was also in accordance with those earlier* in vivo *studies. In those studies, significant effects on hemato-/immunologic parameters were seen with mancozeb doses that ranged from 250 to 1500 mg/kg BW [38] or with cypermethrin (through oral or intraperitoneal routes) at doses ranging from 25 to 300 mg/kg BW [34, 35]. Still, while these* in vitro* studies demonstrated effects from direct exposure of PBMC to each pesticide, questions about relevance of the doses used (0.45–30 *μ*M of parent compounds) seem to persist. Occupational exposure to pyrethroids and ethylenebisdithiocarbamates often results in increases in body burdens of the toxicants that are, in turn, reflected at mg levels of select metabolites of the parent agent (for cypermethrin, 3-phenoxy-benzoate; for mancozeb, ethylene thiourea) in the blood/urine of the workers [17, 39, 40]. As such, at even just a single mg of each metabolite, this would yield corresponding levels of 4.7 (3-phenoxybenzoate) and 9.8 (ethylene thiourea) *μ*M in the human blood/urine of the exposed workers. Thus, the doses of cypermethrin or mancozeb used in the current experiment were likely to have been on par with levels of the parent + metabolite found in workers routinely exposed to either pesticide. Nevertheless, the true relationship between cumulative quantities of metabolites produced by continuous exposures to these pesticides in workers can and should be estimated by others using mathematical toxicokinetic modeling to better estimate at what doses these pesticides would be unquestionably relevant in this type of* in vitro* studies performed here.

## 5. Conclusions

The present study demonstrates that pesticides that are indiscriminately used might increase the risk for immunomodulation in an exposed host. As such, this also reflects the need for continued evaluation of the immunotoxic potentials of these and other types of commonly encountered occupational/environmental chemicals. As the data here also show,* in vitro* studies using freshly obtained human cells (i.e., PBMC) to assess agent-induced changes in endpoints like lymphocyte proliferation and T_H_1/T_H_2 cytokine production will certainly contribute to a better understanding of the effects of xenobiotics on immune function and also increase the arsenal that investigators can use when designing/developing testing regimens to assess such effects.

## Figures and Tables

**(a):** 

Mancozeb (*µ*M)	Proliferation	TNF-*α*	IFN-*γ*	IL-2	IL-10	IL-6	IL-4
Solv.	100.0 ± 0.0	696.4 ± 60.2	1303.4 ± 175.1	56.6 ± 10.9	37.7 ± 7.2	597.8 ± 63.6	19.7 ± 3.1
0.45	105.4 ± 2.5	657.4 ± 69.3	1912.3 ± 127.6	109.4 ± 12.9	41.0 ± 7.3	628.8 ± 69.1	22.2 ± 4.9
0.9	119.9 ± 4.5	621.1 ± 66.9	2401.0 ± 113.0	138.1 ± 23.5	39.8 ± 8.3	551.2 ± 86.1	19.6 ± 4.6
1.87	138.4 ± 6.8	424.7 ± 21.7	2617.8 ± 165.6	152.9 ± 22.2	33.6 ± 8.7	390.6 ± 100.4	17.4 ± 3.1
3.75	162.5 ± 4.6	238.6 ± 35.8	2974.6 ± 411.6	163.7 ± 26.6	24.2 ± 9.6	217.5 ± 72.2	14.5 ± 2.7
7.5	174.5 ± 5.2	129.1 ± 25.2	3539.2 ± 103.7	167.3 ± 28.6	15.1 ± 4.6	107.7 ± 34.9	10.0 ± 1.7

**(b):** 

Cypermethrin (*µ*M)	Proliferation	TNF-*α*	IFN-*γ*	IL-2	IL-10	IL-6	IL-4
Solv.	100.0 ± 0.0	426.6 ± 44.4	2555.9 ± 281.0	127.4 ± 6.2	31.9 ± 5.8	614.5 ± 90.8	17.9 ± 3.2
1.87	102.7 ± 1.4	412.0 ± 22.8	2450.4 ± 352.0	138.4 ± 8.9	28.9 ± 4.9	629.0 ± 31.0	16.2 ± 2.8
3.75	102.7 ± 3.4	409.6 ± 54.3	2421.8 ± 244.5	148.6 ± 9.8	25.9 ± 4.4	601.2 ± 27.0	15.3 ± 2.5
7.5	101.1 ± 1.2	397.8 ± 59.0	2316.2 ± 310.4	145.3 ± 7.9	25.9 ± 2.8	565.9 ± 30.4	13.6 ± 1.8
15	100.0 ± 1.1	382.4 ± 57.0	2222.3 ± 336.4	149.6 ± 13.4	24.3 ± 3.2	538.2 ± 33.9	13.0 ± 1.4
30	98.0 ± 0.8	365.1 ± 42.5	2134.9 ± 364.2	159.6 ± 12.8	23.0 ± 3.3	527.3 ± 34.1	12.1 ± 1.2
